# AAV capsid sites breakdown: large protein insertions impact on vector dynamics

**DOI:** 10.3389/fbioe.2026.1862682

**Published:** 2026-07-03

**Authors:** Mariana V. Ferreira, Marina Curto, André Nascimento, Micael C. Freitas, Pedro M. F. Sousa, Filipe Freire, Tiago M. Bandeiras, Cristina Peixoto, Ana Sofia Coroadinha

**Affiliations:** 1 iBET-Instituto de Biologia Experimental e Tecnológica, Oeiras, Portugal; 2 Instituto de Tecnologia Química e Biológica António Xavier, Universidade Nova de Lisboa, Oeiras, Portugal

**Keywords:** AAV particle characterization, AAV vector, capsid engineering, labeling of AAV particles, large protein insertion, mosaic vectors, rational design

## Abstract

**Introduction:**

Adeno-associated virus (AAV) vectors are one of the most used gene delivery systems, and several capsid engineering strategies have been followed to further improve their performance. Current capsid design approaches predominantly rely on small peptide insertions (<100 a.a.). Integrating large proteins poses challenges due to the nature of protein folding and the complexity of capsid assembly.

**Methods:**

This study seeks to explore capsid engineerable hotspots for large protein insertion, aiming to unlock the use of AAV vectors to deliver large protein cargo and extend their use beyond traditional AAV gene therapy approaches. In this work, we employed a capsid mosaic approach, integrating a mCherry protein (236 a.a) into the VP1 capsid protein (453 or 587 residues) and the N-terminal of VP2 (corresponding to 138 residue of VP1). A systematic breakdown of the different capsid insertions was performed to elucidate insertion site versatility and behaviour.

**Results and Discussion:**

The engineered AAV2/mCherry mosaic vectors presented no major impairments in biophysical and particle functionality. Moreover, alterations in particle titer or biophysical properties were associated with the development of mosaic vectors, rather than the mCherry insertion. In vitro transduction studies showed residues 138 and 453 as particularly permissive sites for functional protein integration without severe structural or functional compromises. While still affecting mCherry fluorescence, both sites preserved the protein’s activity more than site 587. Our work unveils the robustness of AAV capsids to host substantial domain insertions without compromising particle production. The characterisation of these particles ultimately indicated that residue 138 is the most flexible site for inserting proteins up to 236 amino acids, followed by residue 453. These findings establish foundational knowledge for broadening the applications of these vectors to deliver large protein cargo, paving the way for the future development of novel AAV vector systems.

## 1 Introduction

Adeno-associated viral (AAV) vectors represent one of the leading platforms for gene delivery. Current research shows that the genetic modification of the AAV capsid further boosts the success of these therapies (Li and Samulski, 2020). AAV capsid engineering consists of modifying the viral capsid of the virus aiming to enhance its transduction, improve or alter tropism, or avoid immune response (Lee et al., 2018). Two main strategies can be used to develop capsid-engineered AAV vectors, either by directed evolution or rational design approaches. Directed evolution mimics the natural selection processes and consequently generates genetic variants with enhanced specificity and advantageous characteristics through the process of random library mutation and tissue *in vivo* selection (Daya and Berns, 2008). Although successes have been achieved with directed evolution, this strategy presents several disadvantages, such as being time-consuming and costly, and most of the time, resulting in low translation between animal-selected variants and humans. Rational design is based on the detailed knowledge of the AAV biology, capsid properties and viral proteins sequences, allowing specific modifications in the AAV capsid to modulate defined properties and functions (Wu et al., 2000; Lochrie et al., 2006; Daya and Berns, 2008). Rational design techniques are mostly used for small peptide insertions into the capsid of different AAV serotypes. Capsid modification strategies also include amino acid point mutations, chemical approaches, and peptide domain insertions. Serotype 2 still consists of one of the most used serotypes for these strategies (Boucas et al., 2009). Due to the AAV capsid structure, specific 3-fold protrusion or 2-fold depression regions are predominantly used for peptide insertions (Stagg et al., 2022). One of the most used sites for AAV2 capsid engineering is position 587 (VP1 numbering). This is due to its location on the capsid at the second-highest capsid protrusion (Boucas et al., 2009), which leads to this site being highly exposed. Moreover, this region is described to be involved in the AAV2 heparin receptor binding due to the localisation of two arginines in residues 585 and 588 (Kern et al., 2003). A portfolio of subsequent studies confirmed the potential of this insertion site for capsid engineering, especially for tropism alteration or enhancement (Perabo et al., 2006). Similar to residue 587, residue 453 is the most prominently exposed region of the viral capsid (Naumer et al., 2012). Peptide insertions at the 453 sites (VP1 numbering) have been successfully performed, mostly to optimise AAV vector transduction or cell retargeting (Boucas et al., 2009; Naumer et al., 2012). Sites 587 and 453 were the first two examples showing that single point mutations of capsid residues or small peptide insertions (up to 4 kDa) can improve target receptor binding of AAV2 vectors (Boucas et al., 2009). Capsid modifications have also been performed on residue 138 (VP1 numbering), the N-terminal of VP2. This AAV2 region is located at a capsid depression, making it less accessible than previously described sites. Nevertheless, insertions at this site, such as designed ankyrin repeat protein (DARPin)-based targeting ligands (typically ≥14–18 kDa) (Gigout et al., 2005) have been successfully made, presenting physical titers and transduction efficiencies comparable to the unmodified vector (Münch et al., 2013).

Despite several methods being developed to insert specific proteins into the capsid of AAVs, their success is highly dependent on the size and features of the specific inserted protein or peptide. Modifying the AAV capsid often results in a significant reduction in both virus physical particles and transducing titers, especially when large proteins are introduced into the AAV capsid (Warrington et al., 2004). The development of mosaic vectors enabled the mitigation of the detrimental effects of capsid modification using larger proteins, such as titer reduction or inefficient particle production (Gigout et al., 2005). Mosaic vectors present protein insertions in only a subset of the VP1, VP2, and VP3 capsid proteins, increasing viral particle production and transducing titers compared with viruses that carry the insertion in all 60 capsid proteins. For the development of AAV mosaic vectors, the expression of the viral VP1/2/3 needs to be separated. This is achieved by generating new plasmids that express only one or two of the capsid proteins. Large proteins are typically inserted only into VP1 (587 residue or 453 residue) or VP2 (138 residues of VP1, corresponding to residue 1 of the N-terminal of VP2) by expressing the modified protein separately and providing the remaining wild-type capsids in trans (Warrington et al., 2004). For the separation of the viral VPs, site-directed mutagenesis of AAV structural proteins, either in their initiation codons or splicing acceptor sites, is performed. Several literature reports have shown different combinations of site-directed mutagenesis for the AAV2 VPs isolation and trans-complementation (Ruffing et al., 1992; Muralidhar et al., 1994; Warrington et al., 2004; Gigout et al., 2005). Capsid mosaic approaches allowed greater flexibility than current strategies in genetically altering the composition of the AAV capsid and helped expand the size range of proteins able to be inserted into the AAV vector (Warrington et al., 2004). There have been a few studies showing moderately sized proteins (≥ 20 kDa) inserted into the vector’s capsid using this approach. In Judd et al. (2012) 16 inserted mCherry into residue 453, demonstrating the successful insertion of a 28 kDa mCherry protein into the surface of the AAV2 capsid without affecting virus infectivity (Judd et al., 2012). In Feiner et al. (2019) 17 inserted a 29 kDa enzyme β-lactamase into residue 587 of the AAV2 capsid, revealing that the enzyme remained functional, but the produced AAVs had a decreased transduction (Feiner et al., 2019). Lastly, the N-terminal of VP2, residue 138, has also been the subject of studies for large protein insertion (Lux et al., 2005; Büning and Srivastava, 2019). In 2004, Warrington et al.13 inserted a 30 kDa GFP protein into the N-terminal of VP2, residue 138, finding that insertions at residue 138 that were exclusively in VP2 had a minimal effect on viral assembly or infectivity13. The development of capsid mosaic vectors resulted in a significant increase in the types of capsid insertions possible in the AAV vectors (Lux et al., 2005; Büning and Srivastava, 2019). Despite all its potential, there is still a lack of understanding of how these different insertion sites affect the inserted protein (≥ 20 kDa) and impact the AAV vector particle’s biophysical properties and functionality.

This work investigates potential hotspots for large protein fusion by doing a systematic characterisation of the different capsid insertions to elucidate insertion site versatility and the impact of modified capsids on AAV vector performance. For that, a 28 kDa mCherry protein was inserted into AAV2 capsid residues 587 (VP1), 453 (VP1) or 138 (N-terminal of VP2), using a capsid mosaic approach. These findings deliver a reference benchmark for new AAV capsid designs that broaden the applications of these vectors to deliver large protein cargo. Ultimately, it prompts the progression of AAV capsid engineering and opens the avenue for a plethora of new applications in AAV gene therapy (Münch et al., 2013).

## 2 Materials and methods

### 2.1 Plasmid construction

The plasmids used for WT AAV vector production (pAAV2_eGFP, pRC2 and pHelper) were described in Ferreira et al. (2023). The pRC2 and pHelper are an AAV helper-free system acquired from Agilent (Santa Clara, CA, USA). A pUC19 expression plasmid (Addgene plasmid #50005) was used as a backbone for all the VP1 and VP2 isolation constructs developed in this work. All double-stranded DNA sequences (gBclocks) were synthesized by Integrated DNA Technologies (Coralville, IA, USA). The backbone for all vector cassettes was developed by inserting a CMV promoter sequence into the multiple cloning site of pUC19 plasmid. The pUC19_CMV was linearized at the HindIII restriction site. Several gBlock coding for VP1 or VP2 with the corresponding missense mutations (as described in the Supplementary Table S1) and followed by a SV40 poly(A) signal, were synthesized and inserted in the previously linear plasmid, ultimately creating the following mosaic constructs: pUC19_CMV_VP1_A; pUC19_CMV_VP1_B; pUC19_CMV_VP1_C; pUC19_CMV_VP2_D; pUC19_CMV_VP2_E. Several gBlock coding for VP1 or VP2 with the corresponding missense mutation and mCherry sequence flanked by GGSG linkers at the VP1_C 453 residue, VP1_C 587 residue, VP2_D 138 (N-terminal) residue and VP2_E_138 residue were also developed. These synthesized sequences were inserted in the previously linear plasmid, generating the following constructs: pUC19_CMV_VP1_C_453_mCherry; pUC19_CMV_VP1_C_587_mCherry; pUC19_CMV_VP2_D_138_mCherry; pUC19_CMV_VP2_E _138_mCherry. For the complementary mosaic constructs the wild-type pRC2 was used, leading to the expression of the wild-type rep proteins and the complementary wild-type VP proteins. For the expression of only the desired VPs a missense mutation was performed using site-directed mutagenesis PCR (as described in the Supplementary Table S1). Creating the following mosaic complementary constructs: pVP2/3_RC; pVP1/3_RC.

### 2.2 Cell lines and culture conditions

Human embryonic kidney 293T (HEK 293T) and HT-1080 human epithelial adherent cells, purchased from ATCC (ATCC-CRL-3216, ATCC-CCL-121 respectively), were cultured and expanded using Dulbecco’s modified Eagle’s medium (DMEM) (Corning™, New York, NY, USA), supplemented with 10% (v/v) fetal bovine serum (FBS) (Gibco™, Thermo Scientific™, Waltham, MA, USA) and maintained at 37 °C in a humidified atmosphere containing 8% CO2.

### 2.3 Transient transfection studies

293T cells were seeded at 1 × 10^5^ cells/cm^2^. The cells were transfected 24 h post-seeding using PEI PRO (PolyPlus, Illkirch, France) at a mass ratio of 1:1 (DNA:PEI) and 1.5 μg of total DNA *per* million cells, each plasmid expression cassette at 0.5 μg. The medium was exchanged 24 h post-transfection to fresh DMEM with 10% (v/v) FBS. After 48 h post-transfection, the cells were harvested, pelleted and reporter protein eGFP expression was measured by Flow Cytometry using BD FACSCelesta (BD Bioscience, NJ, USA), for transfection efficiency evaluation.

### 2.4 AAV vector production

For AAV2 vector production, HEK 293T cells were seeded at 8 × 10^4^ cells/cm^2^. After 24 h, using the transfection reagent PEIpro (PolyPlus, IIIkirch, France), the cells were transfected by triple transient transfection at a mass ratio of 1:1 (DNA:PEIpro) and 2.0 μg of total DNA for 10^6^ cells, each plasmid expression cassette at 0.5 μg. Again, we used a pHelper plasmid and pITR_CAG_eGFP as transgene on all productions. The third plasmid used varied according to the tested condition, and the last one was the plasmid that allowed to complement the VP in testing with the other two VPs and with *rep* gene (pAAV_VP1_A or pAAV_VP1_B or pAAV_VP1_C + pAAV_VP2/3_RC) (pAAV_VP2_D or pAAV_VP2_E + pAAV_VP1/3_RC). After 24 h post-transfection, the culture medium was replaced with fresh DMEM containing 10% (v/v) FBS. Cell harvest was performed at 72 h post-transfection and transfection efficiency was evaluated by flow cytometry, using BD FACSCelesta (BD Bioscience, NJ, USA), measuring eGFP protein expression. Following the harvest process, the cells were centrifuged for 10 min at 300 × g, washed with DPBS and resuspended in a lysis buffer containing 20 mM Tris-HCL, pH 7.5, 150 mM NaCl, 10 mM MgCl_2_, 1% TritonX-100 (v/v), 1Xprotease inhibitor cocktail (Roche, Basel, Switzerland), and 50 U/mL of Benzonase (Merck, Darmstadt, Germany). Lysis of the cells was performed at 37 °C for 45 min with subtle agitation. Subsequently, the lysate was centrifuged for 10 min at 140,00 × g at 4 °C and the AAV2 supernatant was retrieved and stored at −80 °C.

### 2.5 AAV vector purification and buffer exchange

AAV vector affinity purification was conducted using the AAVPro Purification kit for AAV2 (TaKaRa, Kusatsu, Shiga, Japan), following the manufacturer’s recommendations and instructions. For long-term preservation of the viruses’ stability at −80 °C, the elution buffer was exchange for DPBS with 0.01% (v/v) of pluronic using Amicon® Ultra 4 mL Filters with 100 kDa cutoff, for Protein Purification and Concentration. This membrane has a high recovery Ultracel® regenerated cellulose membrane. Firstly, the Amicon membrane was washed with 4 mL of DPBS with 0.01% (v/v) of pluronic and centrifuged for 2 min at 4,000 × g allowing all the buffer to completely flow-thought. This process was repeated four times, until 3 mL of the AAV2 vectors were added to the filter. The vector samples were washed up to four times by adding 2 mL of DPBS with 0.01% (v/v) of pluronic and centrifuged 2 min at 2000 g, each time, not allowing all the buffer to flow-thought. Up to 2 mL of AAVs in DPBS with 0.01% (v/v) pluronic were retrieved from each sample and then store at −80 °C.

### 2.6 AAV2 *in vitro* transduction

HT-1080 cells were seeded at 3.5 × 10^4^ cells/cm^2^, in 24-well plates. Following 24 h after the seeding, the transduction was performed by removal of the medium and transducing the cells with 0.2 mL of purified AAV vector samples at a vector dose (multiplicity of infection (MOI)) of 1 × 10^4^ V.G./cell. The vector doses were diluted in DMEM supplemented with 2% (v/v) FBS. Fresh medium was added 24 h post-transduction (DMEM with 10% (v/v) FBS). The transduction results were assessed by flow cytometry using BD FACSCelesta (BD Bioscience, NJ, USA) measuring eGFP and mCherry protein expression at diverse time points (2, 4, 6, 8, 12, 24, 48, and 72 h post-transduction).

### 2.7 Analytical quantification methods

#### 2.7.1 Total particle quantification (T.P.)

The determination of total AAV2 total particle concentration (T.P.) was performed using the AAV2 ELISA assay (Progen Biotechnik GMBH, Heidelberg, Germany) according to the manufacturer’s instructions. The absorbance was quantified at 450 nm on an Infinite PRO NanoQuant (Tecan, Männedorf, Switzerland) microplate multimode reader using a clear 96-well plate provided in the kit. The samples were quantified using multiple dilutions.

#### 2.7.2 Vector genome copies quantification (V.G.)

DNA extraction was performed according to the instructions in the High Pure Viral Nucleic Acid Kit (Roche, Basel, Switzerland) manual and was followed by real-time qPCR. Determination of the number of viral DNA copies was performed using LightCycler® 480 SYBR Green I Master (Roche Applied Science, Penzberg, Germany) according to the manufacturer’s instructions on a LightCycler® 480 Real Time PCR System (Roche Applied Science). The primers used were Fw (5′-ACT​GTG​TTT​GCT​GAC​GCA​AC-3′) and Rv (5′-ACA​ACA​CCA​CGG​AAT​TGT​CA-3′) targeting the Woodchuck hepatitis PRE (WPRE). The reference standard used was a linear pAAV2_frGFP_myc_HPRT1 plasmid (Ferreira et al., 2023). Plasmid linearization was performed with ScaI enzyme within the blasticidin gene. Serial dilutions from 1 × 10^8^ copies *per* μL were performed.

#### 2.7.3 Transduction units quantification (T.U.)

HT-1080 cells were seeded at 3.5 × 10^4^ cells/cm^2^, in 24-well plates. After 24 h, the cells were transduced by removing the medium and infecting the cells with 0.2 mL of viral supernatants at serial dilutions (ranging from 1:100 to 1:384,000), performed in fresh DMEM supplemented with 2% (v/v) FBS. Fresh medium (DMEM with 10% (v/v) FBS) was added 24 h post-transduction. The cells were harvested and analysed 48 h after transduction for eGFP fluorescence by flow cytometry using BD FACSCelesta™ (BD Bioscience, NJ, USA).

### 2.8 Total protein extraction and quantification

The cells were harvested, counted, pelleted at 300 × g for 10 min. Cell lyses was performed using Mammalian Protein Extraction Reagent (M-PER) (Thermo Scientific™) with 1x cOmplete™ EDTA free Protease Inhibitor Cocktail (Roche Applied Science), using 100 μL *per* 2 million of cells. The mixture was vortexed and placed at 4 °C for 10 min. Extracts were clarified by centrifugation (140,00 × g for 10 min). Samples were stored at −20 °C for short-term and −80 °C for long-term. The total protein content was quantified using a BCA Protein Assay Kit (Thermo Fisher Scientific). Samples were applied in serial dilutions and reference material was applied in duplicate. The absorbance was measured with an Infinite PRO NanoQuant (Tecan) microplate reader.

### 2.9 Capillary Western blot

The Jess™ Simple Western system (ProteinSimple, San Jose, CA, USA) is an automated capillary-based size separation system that can detect chemiluminescence and fluorescence signals. To verify the VPs constructs, the manufacturer’s standard method was followed for the 12–230-kDa Jess separation module (SM-W004). The primary antibodies used were: Anti-AAV VP1 VP2 VP3 (Progen, Biotechnik GMBH, Heidelberg, Germany) with 1:50 dilution, to detect the AAV2 viral proteins; anti-rabbit monoclonal anti-mCherry (R&D Systems, Minneapolis, Minnesota, EUA) antibody with 1:50 dilution to detect mCherry protein; and a monoclonal antiβ-actin (Aldrich, St. Louis, MO, USA) antibody produced in mouse was used as a loading control, diluted 1:50 in all samples. The secondary antibodies used were specific for this system: anti-mouse secondary antibody (042–205), anti-rabbit secondary antibody (042–206), anti-mouse NIR detection module for Jess (DM-009). The Anti-AAV VP1/VP2/VP3 and anti-mCherry tag detections was performed by chemiluminescence and β-actin by fluorescence. The Compass Simple Western software 6.2.0 (Protein Simple, CA, USA) was used to generate a digital image of the chemiluminescence and fluorescence capillary run. The software also automatically estimated the heights (chemiluminescence and fluorescence intensity), area, and signal/noise ratio of each sample. These results were assessed as electropherograms demonstrating the peak of chemiluminescence intensity with a simulated lane view. By using the peak area, protein quantification could also be performed.

### 2.10 Flow cytometry

Fluorescence analysis of the reporter proteins, GFP present in the AAV vector expression cassette and mCherry protein in the AAV2 vector capsid, was conducted using a BD FACSCelesta (BD Bioscience, NJ, USA). The cells were harvest in DPBS with 2% (v/v) of FBS. Data analysis of the results was carried out using FlowJo software v10.9 (BD Bioscience, NJ, USA). A density plot using FSC-H and SSC-A was first employed to distinguish live and death cells. Among the live cells, a second density plot using FSC-A and FSC-H was implemented for selection of single cells. These single cells were then analysed for mCherry and GFP fluorescence using the lasers yellow-green and blue, respectively. An internal quality control of the equipment was performed, in every experiment, using rainbow beads to guarantee that the fluorescence intensity measurements remained comparable throughout the experiments.

### 2.11 AAV biophysical characterization

#### 2.11.1 VPs ratios

The Jess™ Simple Western system was used as described previously. Purified AAV particles were loaded at three different concentrations (1 × 10^8^ T.P; 5 × 10^8^ T.P; 1 × 10^9^ T.P) to determine the average of the VPs ratios. The primary antibodies used were: Anti-AAV VP1, VP2, and VP3 (Progen, Biotechnik Gmbh, Heidelberg, Germany), diluted 1:50, to detect the AAV2 viral proteins. By using the peak area, protein quantification was performed.

#### 2.11.2 Heparin biding

AAv vectors binding to heparin was measured using biolayer interferometry using OctetRED96 system (Sartorius, Gottingen, Germany) high precision streptavidin (SAX) biosensors and heparin-biotin (Creative PEGWorks, North Carolina, USA). AAV vectors were loaded and measured as described for SAX biosensors and receptors, using the following assay parameters: 60s baseline step, 300s loading step, 300s quenching step, 60s baseline step, 1000s association step, 500s dissociation step, all in 400 rpm shake speed. Heparin and biotin were used at a concentration of 25 μg/mL. Data were acquired in kinetics mode and analyzed using the Data Analysis software v9.0 (FortéBIO, Pall Corp., Port Washington, NY, USA).

#### 2.11.3 Thermostability

Nano Differential Scanning Fluorimetry (nDSF) was performed using Prometheus NT.48 (NanoTemper Technologies, München, Germany). Samples were loaded in nanoDSF-grade high-sensitivity capillaries (NanoTemper Technologies GmbH, München, Germany) and exposed to thermal stress from 20 °C to 95 °C by thermal ramping rate of 1 °C/minute. For the comparative analysis all samples were loaded at 10^12^ T.P./mL. The intrinsic protein fluorescence at 330 and 350 nm, as well as light scattering, were recorded. Thermal stability parameters, such as the melting temperature (Tm) derived from fluorescence at 330 nm, were calculated using PR.ThermControl software (NanoTemper Technologies, München, Germany).

#### 2.11.4 Negative stain transmission electron microscopy

Negative staining transmission electron microscopy (TEM) was employed after purification to ensure the purity and integrity of the capsids. The samples were first fixed by adding 2% (v/v) formaldehyde in 0.05 M Phosphate buffer and fixed for 1 h at room temperature. 3 μL of AAVs were applied onto a glow-discharged 100 mesh copper grid coated with 1% (w/v) formvar in chloroform and carbon. The AAVs were incubated for 10 min before washing 10 times for 10 s in distilled water The grid was then stained twice in 2% uranyl acetate (w/v) for 5 min before blotting dry. Grids were imaged on a transmission electron microscope (FEI Tecnai G2 Spirit BioTWIN) operated at 120 kV equipped with a CCD Camera (Olympus-SIS Veleta).

### 2.12 Statistical analysis

The data were expressed as mean ± SD and analyzed with GraphPad Prism 8.0.1 software (San Diego, CA, USA). Statistical significance was determined using one-way ANOVA followed by Tukey’s *post hoc* multiple comparison test.

## 3 Results

### 3.1 Capsid mosaic approach

**FIGURE 1:**
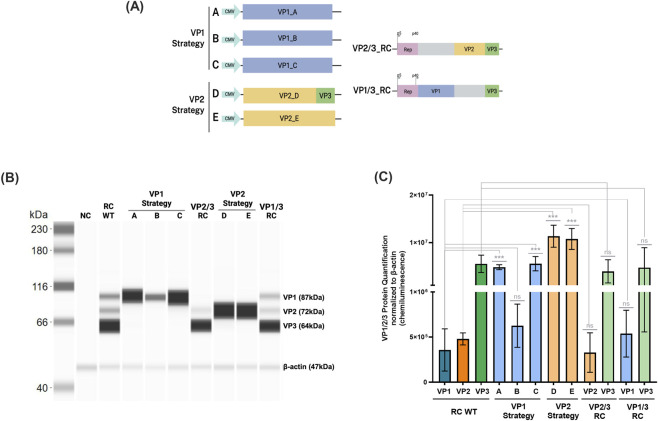
Validation of AAV2 mosaic constructs. Validation of mosaic constructs was performed by transiently transfecting 293T cells with the plasmids: VP1(A/B/C)/VP2(D/E)/VP2/3_RC/VP1/3_RC and a GFP-expressing plasmid. Transfection efficiency was evaluated by flow cytometry at 48 h (data not shown). **(A)** Schematic representation of mosaic plasmid constructs; A-expresses VP1, by mutating a splicing acceptor 2,228; B- expresses VP1, by mutating VP2 and VP3 start codon; C- expresses VP1, by mutating a splicing acceptor 2,228 and start codon of VP2 and VP3; D-expresses VP2, by mutating a start codon of VP1; E-expresses VP2, by mutating a start codon of VP1 and VP3; VP2/3_RC- expresses VP2 and VP3 in the rep/cap backbone; VP1/3_RC- expresses VP1 and VP3 with rep/cap backbone. **(B,C)** Capillary Western blot analysis using cell extracts of VP1/2/3 viral proteins individually transfected. Cell extracts were analyzed by immunoblotting with anti-VP1/2/3 antibody and anti-β-actin.VP1 (87 kDa), VP2 (72 kDa), VP3 (62 kDa) and β-actin (47 kDa) were detected by chemiluminescence channel. **(B)** WB compass software 6.2.0 (ProteinSimple, CA, USA) was used for simulation of a standard polyacrylamide gel run. **(C)** Quantification of the viral proteins. NC- Negative control; RC WT-rep/cap wild type. All data represent the mean ± SD; *n* = 3; ns—no significance; ****p < 0.0001 by Tukey’s *post hoc* multiple comparison test, relative to the corresponding wild type VPs, as indicated by the connecting lines. The illustrations were created with BioRender.com accessed on the 15 May 2025.

The insertion of larger proteins into the AAV capsid often results in a significant reduction in viral particles and transducing titers. Mosaic vector approaches can help overcome these issues by allowing the protein insertions in only a subset of the VP1, VP2, and VP3 capsid proteins. This strategy can ultimately help sustain good viral particle production and transducing titers compared to insertions in all 60 capsid proteins. For that, separated VP expression cassettes were developed (Figure 1; Supplementary Table S1). The isolations of viral proteins were done with missense mutations according to previous work described in the literature (Muralidhar et al., 1994; Warrington et al., 2004; Guo et al., 2012). All the developed mosaic vector constructs were validated to determine the correct isolated VPs protein expression. This was assessed by Western Blot analysis, where the protein expression and corresponding molecular weight were determined. Additionally, it is critical to ensure whether the modifications introduced in the new mosaic AAV2 vectors influence their production efficiency and functional performance. To this end, mosaic vectors were generated, and their transducing units, viral genome titers, and total particle counts were quantified.

#### 3.1.1 Mosaic construct validation

Initial validation of the new constructs that expressed only one or two of the capsid proteins was performed by transient transfection of the different VP constructs previously described (Figure 1A; Supplementary Table S1). To assess the transfection efficiency of all conditions, a GFP-expressing plasmid was added to each transfection mix. Viral protein production and GFP expression were evaluated at 48 h post-transfection by Capillary Western blot and flow cytometry, respectively. All conditions demonstrated a similar transfection efficiency exceeding 90% (data not shown), allowing for an accurate comparison between conditions. Capillary Western blot analysis of the cell extracts allowed the successful validation of the new constructs, as the separated VPs presented the corresponding molecular weights. No leakage expression of the silenced VPs was detected, with the exception of construct VP1/3_RC, where VP2 is detected (Figure 1B). Protein quantification results (Figure 1C) showed that for VP1 separation, the A and C strategies rendered up to a 16-fold higher protein production when compared to the WT condition. In contrast, the B strategy presented 9-fold less VP1 production than strategies A and C. For VP2 separation, both strategies (D and E) presented similar results with up to 24-fold higher VP2 production than the WT. Overall, the strategies of VP1 and VP2 isolation, where CMV was used, rendered higher protein production than WT VP1 and VP2, respectively. The complementary constructs VP2/3_RC and VP1/3_RC showed similar levels of VP protein production as the WT construct. This is in line with what was expected, as the expression of these VPs is controlled by the WT AAV p40 promoter.

#### 3.1.2 Evaluation of the production of AAV2 mosaic vectors

**FIGURE 2:**
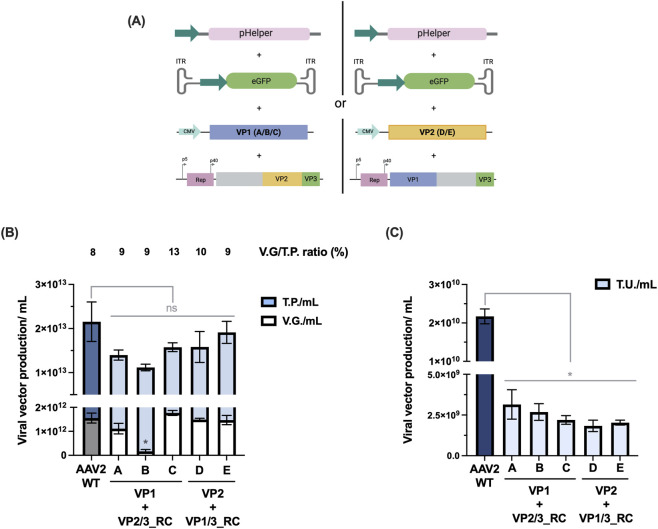
Characterization of the AAV2 vectors production. AAV mosaic vectors were produced in 293T cells after transient transfection of the corresponding plasmids: VP1(A/B/C) + VP2/3_RC or VP2(D/E) + VP1/3_RC; GFP expression plasmid and Helper plasmid. The vectors were harvested at 72 h post-transfection. Produced AAV vectors were quantified by viral genome particles (V.G), total particles (T.P) and transducing units (T.U.) by *in vitro* titration assay in HT-1080 cells. **(A)** Representation of the plasmid combination used for mosaic vector production. The grey rectangle represents the VP region that is not expressed due to the introduced mutation. **(B)** Results of T.P. and V.G. quantifications *per* mL. Viral quality is shown as the V.G./T.P. ratio (%), which indicates the proportion of genome-containing particles relative to total particles (empty and full ratios). **(C)** Results of T.U./mL. NC- Negative control; A-expresses VP1, by mutating a splicing acceptor 2,228; B- expresses VP1, by mutating VP2 and VP3 start codon; C- expresses VP1, by mutating a splicing acceptor 2,228 and start codon of VP2 and VP3; D-expresses VP2, by mutating a start codon of VP1; E-expresses VP2, by mutating a start codon of VP1 and VP3; VP2/3_RC- expresses VP2 and VP3 with *rep*; VP1/3_RC- expresses VP1 and VP3 with *rep*. All data represent the mean ± SD; *n* = 3; ns—no significance; *p ± 0.046 by Tukey’s *post hoc* multiple comparison test. The illustrations were created with BioRender.com accessed on the 15 May 2025.

The production and functionality assessment of AAV2 mosaic vectors was performed by transfecting HEK 293T cells with four plasmids *per* condition. The pHelper and eGFP transfer plasmid were used in all transfection conditions, together with the isolated VP constructs under study, plus the two complementary VPs constructs also expressing the *rep* genes (Figure 2A). The results were analyzed by flow cytometry at 72 h post-transfection, when all conditions presented transfection efficiency above 90% (data not shown). Vector titers were quantified for total particles, viral genome particles and transducing units (Figures 2B,C). The ratio of viral genome particles and total particles was also determined.

All mosaic vectors presented similar transduction units’ titers between them, but overall, 8-fold lower than the WT AAV2 vectors (Figure 2C). All conditions also presented similar results for V.G. titers spanning from 6.2 × 10^11^ and 1.8 × 10^12^ V.G./mL, with the exception of mutation B_VP1, which presented the lowest titers of 1 × 10^11^ V.G./mL. When comparing total particles, a similar trend to V.G. was observed. In contrast, mutations C_VP1, D_VP2 and E_VP2 presented similar titers (V.G./mL and T.P./mL) as the WT AAV2 control. Mutation A_VP1 performed in between the latter and B_VP1. Overall, the development of the mosaic vectors was successful, as the results showed that all mosaic vectors had a correct particle assembly, genome incorporation, and the ability to transduce the target cells.

### 3.2 mCherry protein insertion into AAV2 mosaic vectors

**FIGURE 3:**
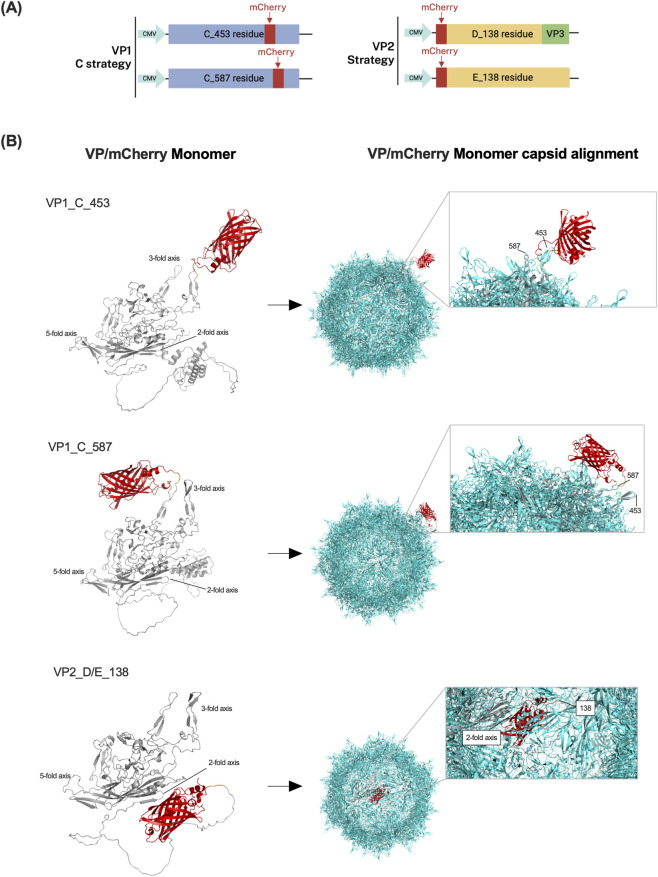
Representation of mCherry constructs. **(A)** Representation of separated viral proteins expressing cassettes with mCherry insertions. C_453 -Expresses VP1, with mCherry inserted into 453 site; C_587- Expresses VP1, with mCherry inserted into 587 site; D_138- Expresses VP2 (and VP3) with mCherry inserted into 138 site; E_138- Expresses VP2, with mCherry insertion on 138 site. The illustration was created using BioRender.com, accessed on 15 May 2025.2025. **(B)** Molecular model of engineered VPS fused with mCherry. On the left, the structures of the monomeric forms of VP1_C_453, VP1_C_587 and VP2_D/E_138 (grey) proteins, containing an inserted mCherry (red), flanked by a GGSG linker (orange), predicted by AlphaFold (Abramson et al., 2024). Symmetry axes are indicated. On the right, a schematic image shows the structural alignment of the theoretically predicted structures of VP/mCherry monomers, represented in the left panel, with the cryo-EM structure of AAV2 WT (cyan, PDB: 8FYW). This representation does not account for VP stoichiometry and shows only one representative VP/mCherry monomer. The representation was performed using the PyMOL Molecular Graphics System, version 3.0, Schrödinger, LLC.

The best three mosaic constructs were chosen for mCherry insertion in the AAV vector capsid: C_VP1, D_VP2, and E_VP2. For VP1, mCherry was inserted into two sites, 453 and 587. These sites are the two most external sites of the capsid, making the fused mCherry easily accessible. For VP2, mCherry was inserted into site 138 using both mutation strategies. The only difference in these mutations is the amount of VP3, which ultimately allows for a better understanding of how the VP3 protein influences the stability and functionality of these engineered vectors. Site 138 is located in the N-terminal of VP2, situated on a depression of the AAV2 capsid, allowing for protein insertions with different structural exposure than the previous sites (Figure 3).

#### 3.2.1 VP-mCherry fusion constructs validation

**FIGURE 4:**
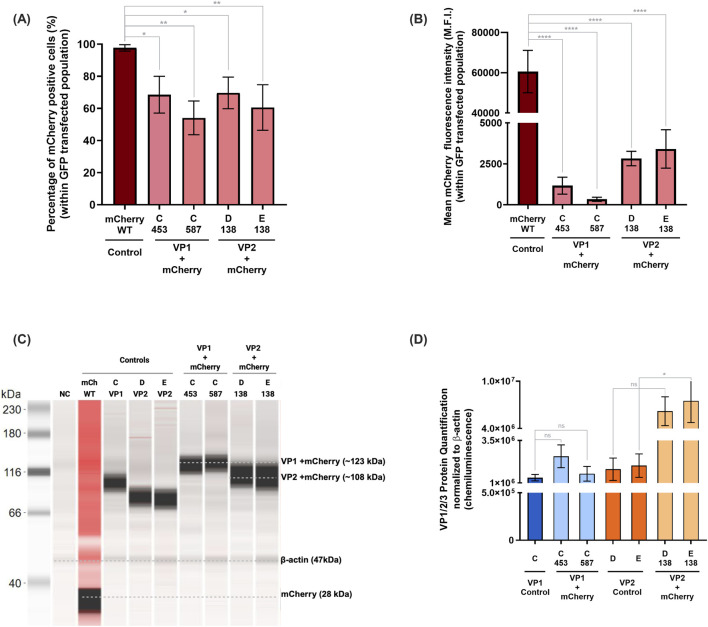
Evaluation of mCherry constructs. **(A,B)** 293T cells were transient transfected with the plasmids: RC WT/mCh WT/C_VP1/C_453/C_587/D_VP2/D_138/E_VP2/E_138 and GFP expressing plasmid. mCherry fluorescence signal was evaluated by flow cytometry at 48 h post-transfection. **(A)** Results of mCherry positive cells within GFP transfected population **(B)** Results of mean fluorescence intensity, measured by flow cytometry, for mCherry, containing constructs at 48 h post-transfection. **(C,D)** Capillary Western blot analysis using cell extracts of VP1/2/3 viral proteins with mCherry insertion. Cell extracts were analyzed by immunoblotting with anti-VP1/2/3 antibody, anti-β-actin and anti-mCherry.VP1 (87 kDa), VP2 (72 kDa), VP3 (62 kDa), β-actin (47 kDa) and mCherry (28 kDa) proteins were detected by chemiluminescence channel. For the mCherry run, β-actin was detected by fluorescence channel. **(C)** WB compass software 6.2.0 (ProteinSimple, CA, USA) was used for simulation of a standard polyacrylamide gel run. **(D)** Quantification of AAV vector viral proteins. NC- Negative control (293T non-transfected cells); mCh WT-mCherry wild type (293T cells transfected with a mCherry expressing plasmid); C_453 -Expresses VP1, with mCherry inserted into 453 site; C_587- Expresses VP1, with mCherry inserted into 587 site; D_138- Expresses VP2 (and VP3) with mCherry inserted into 138 site; E_138-Expresses VP2, with mCherry insertion on 138 site; VP2/3_RC- expresses VP2 and VP3 with *rep*; VP1/3_RC- expresses VP1 and VP3 with *rep*. All data represent the mean ± SD; *n* = 3; ns-no significance; *p ± 0.020; **p ± 0.0043; ****p < 0.0001 by Tukey’s *post hoc* multiple comparison test.

Initial validation of the new constructs with the mCherry fusion in viral proteins VP1 or VP2 was performed by transient transfection in HEK 293T cells. To assess the transfection efficiency of all conditions, a GFP-expressing plasmid was used (Supplementary Figure S1). The results were assessed by flow cytometry and Capillary Western blot, 48 h post-transfection. All conditions presented a transfection efficiency above 80% (data not shown). All the VP-mCherry constructs showed functional mCherry fusions, as all insertion sites presented similar percentages of mCherry positive cells, above 60% (Figure 4A). Higher differences were observed in the fluorescence intensity of the fused mCherry protein, which allowed to compare its activity when fused to the different insertion sites. In Figure 4B, differences in mCherry fluorescence intensity are shown between the different insertion sites on the VPs and the WT non-fused mCherry control (a mCherry expressing plasmid transfected in 293T). The mCherry insertions into VP1, C_453 and C_587 presented a 43 and 148-fold decrease, respectively, in mCherry fluorescence intensity compared to the control. While in VP2 fusions mCherry fluorescence intensity only decreased 16-fold. Nevertheless, insertion sites 453 and 138 still presented mCherry fluorescence intensity values above 1000 MFI (A.U.), indicating that fused mCherry is functional.

To assess the protein expression of the VP-mCherry constructs, a capillary Western blot was performed where viral and mCherry proteins from cell extracts were detected (Figures 4C,D). Capillary Western blot results showed a shift in molecular weight in both samples of VP1 and VP2 with mCherry insertions when compared to their respective VP1 and VP2 controls. This shift, around 30 kDa, corresponds to the molecular weight of mCherry protein, detected at 28 kDa. Thus, validating the mCherry fusion to the correspondent VPs. Using the Capillary Western blot software analysis, the area of the peak of the VP was analysed. These values were used to quantify the protein being produced for all four mCherry insertions and their respective isolated VPs controls, as shown in Figure 4D. Although VP1 insertions showed similar results to respective VP1 control (VP1_C), VP2 insertions rendered a higher protein production, presenting a 4-fold increase in VP2/mCherry protein production compared to VP2 controls (VP2_D and VP2_E).

#### 3.2.2 AAV2/mCherry vector production

**FIGURE 5:**
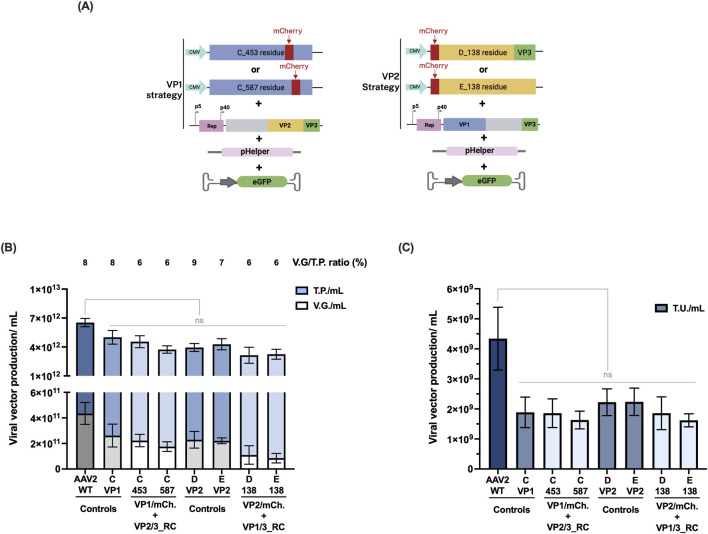
Evaluation of the AAV2/mCherry mosaic vectors production. AAV mosaic vectors with mCherry insertion were produced in 293T cells after transient transfection of the corresponding plasmids: VP1+mCherry (C_453 or C_587) + VP2/3_RC or VP2+mCherry (D_138/E_138) + VP1/3_RC, helper plasmid and GFP transfer expression plasmid. The vectors were harvested at 72 h post-transfection. Produced AAV vectors were quantified by transducing units (T.U.), viral genome particles (V.G) and total particles (T.P) by *in vitro* titration assay in HT-1080 cells, RT-qPCR and AAV2 ELISA, respectively. **(A)** Representation of the plasmid combinations for the AAV2/mCherry production. The grey rectangle represents the VP region that is not expressed due to the introduced mutation. **(B)** Results of T.P. and V.G. quantifications *per* mL. Viral quality is shown as the V.G./T.P. ratio (%), which indicates the proportion of genome-containing particles relative to total particles (empty and full ratios). **(C)** Results of T.U./mL. C_VP1- control only expressing VP1; C_453 -Expresses VP1, with mCherry inserted into 453 site; C_587- Expresses VP1, with mCherry inserted into 587 site; D_138- Expresses VP2 (and VP3) with mCherry inserted into 138 site; E_138-Expresses VP2, with mCherry insertion on 138 site; VP2/3_RC- expresses VP2 and VP3 with *rep*; VP1/3_RC- expresses VP1 and VP3 with *rep*. All data represent the mean ± SD; *n* = 3; ns-no significance by Tukey’s *post hoc* multiple comparison test. The illustrations were created with BioRender.com accessed on the 15 May 2025.

After validation of the mCherry fusion to the mosaic vector constructs, the newly engineered AAV2/mCherry vectors were produced and quantified. These vectors were produced in HEK 293T cells using a GFP-expressing transfer plasmid, a helper plasmid, each of the fused mCherry/VPs constructs and the corresponding complementary VP_RC constructs (Figure 5A). The produced AAV2/mCherry engineered particles were harvested at 72 h post-transfection, and transfection efficiency was evaluated by flow cytometry at the same time point. All conditions presented a transfection efficiency above 90% (data not shown). To evaluate the vectors’ production efficiency, viral genome titers, total particles and transducing units were quantified. Vector production titers were compared to WT AAV2 and AAV2 mosaic vectors to assess the production efficiency of the engineered AAV2/mCherry vectors (Figures 5B,C).

No major loss in T.P./mL total particles titers was observed, as all AAV2/mCherry vectors maintain the same range of titers of 10^12^ T.P./mL, similar to the controls. In terms of viral genome particles (V.G./mL), all mosaic AAV2 vector controls and AAV2/mCherry engineered vectors presented less than a 5-fold decrease, with no statistical significance. Regarding functional particles (T.U./mL), all mosaic AAV2 and engineered AAV2/mCherry vectors showed a decrease when compared to WT AAV2. Yet, they presented titers up to 1 × 10^9^ T.U./mL. All vectors, including WT control, presented similar quality between 6 and 9% full particles, showing that viral genome incorporation was not affected by mCherry capsid fusion.

#### 3.2.3 AAV2/mCherry vector purification

**FIGURE 6:**
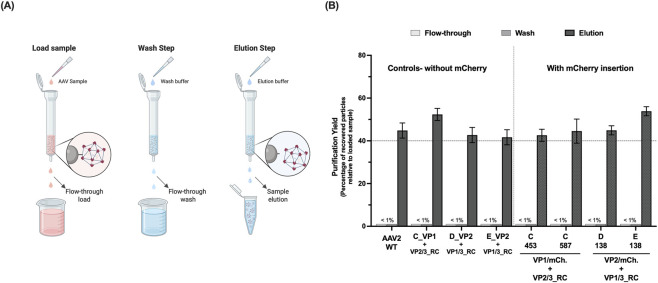
Evaluation of the AAV2/mCherry vectors downstream purification yields. AAV vectors were produced in 293T cells and harvested at 72 h post-transfection. The vectors were purified using AAVX magnetic beads. The affinity purification yield of each AAV vector was assessed using the T.P./mL titer calculated by ELISA. **(A)** Schematic representation of the affinity purification process. **(B)** Purification yields. Results are shown as the percentage of recovered vector in each purification step: flow-through of AAV2; washing step and elution step in which AAV vectors were recovered. C_453 -Expresses VP1, with mCherry inserted into 453 site; C_587- Expresses VP1, with mCherry inserted into 587 site; D_138- Expresses VP2 (and VP3) with mCherry inserted into 138 site; E_138-Expresses VP2, with mCherry insertion on 138 site; VP2/3_RC- expresses VP2 and VP3 with *rep*; VP1/3_RC- expresses VP1 and VP3 with *rep*. All data represent the mean ± SD; *n* = 2. The illustrations were created with BioRender.com accessed on the 15 May 2025.

Both *in vivo* and *in vitro* preclinical gene therapy studies require highly purified AAV preparations (Guo et al., 2012; Nass et al., 2017). Affinity purification is one of the most used methods, allowing for selective purification of AAV vectors. The engineered AAV2/mCherry particles were purified using a CaptureSelect AAVX affinity resin containing a VHH affinity ligand that recognises conformational determinants on the intact viral capsid (Thermofisher, 2026). For each purification step, the T.P. titers were quantified and used to assess the recovery yields (Figure 6A). Figure 6B shows the result of the purification on the quantity of vector retrieved from the column after each purification step. For the first two steps, loading and washing, almost no AAV2 vectors were detected in these two fractions in all conditions. All the controls presented an average recovery of 40%–50% in the elution step. The same was observed for the engineered AAV2/mCherry particles with a recovery yield of ≥40%. Overall, the efficiency of the purification processes remained similar between control and engineered AAV2/mCherry conditions, showing that the insertion of a large protein into the AAV2 capsid did not impact the purification of the vectors when using CaptureSelect AAVX affinity ligand. Additionally, this avoids the need to modify the particle purification method. Alternative methods, such as cesium chloride gradient ultracentrifugation, can separate viral particles based on density but are time-consuming, yield low quantities, and may still contain significant protein and DNA impurities (Rayaprolu et al., 2013).

#### 3.2.4 mCherry functional assessment by *in vitro* transduction

**FIGURE 7:**
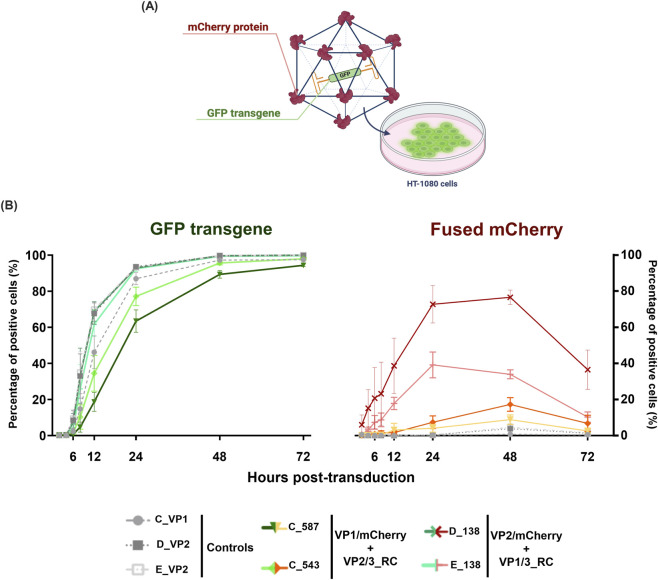
Evaluation of the transduction profile of engineered AAV2/mCherry vectors. Purified AAV2/mCherry engineered vectors were used to transduce HT-1080 cells at a vector dose of 1 × 10^4^ V.G./cell. The results were evaluated by flow cytometry at various time points: 2, 4, 6, 8, 12, 24, 48 and 72 h post-transduction. **(A)** Schematic representation of the engineered AAV2/mCherry vector. **(B)** Percentage of positive cells through transduction. All three controls are represented with grey bars. On the left, all green bars correspond to expression of eGFP. And on the right, all red bars correspond to expression of the mCherry protein, fused to the AAV2 capsid. At 48 h post-transduction, only AAV2/mCherry_C_587 vectors showed a 1.5-fold lower GFP signal with a significance of ****p < 0.0001 using Tukey’s *post hoc* multiple comparison test. AAV2/mCherry_D_138 vectors mCherry signal compared to the other engineered vectors shows a significance of ****p < 0.0001 using Tukey’s *post hoc* multiple comparison test. C_VP1- control only expressing VP1; C_453 -Expresses VP1, with mCherry inserted into 453 site; C_587- Expresses VP1, with mCherry inserted into 587 site; D_138- Expresses VP2 (and VP3) with mCherry inserted into 138 site; E_138-Expresses VP2, with mCherry insertion on 138 site; VP2/3_RC- expresses VP2 and VP3 with *rep*; VP1/3_RC- expresses VP1 and VP3 with *rep*. All data represent the mean ± SD; *n* = 3. The illustrations were created with BioRender.com accessed on the 15 May 2025.

To gain insight into how large protein insertions affect viral particle functionality, the transduction profile was evaluated. Moreover, an *in vitro* transduction measuring mCherry percentage of positive cells allowed to understand the fused mcherry protein activity and decay, in the context of AAV transduction. Furthermore, the GFP positive cells were also measured to assess the transgene expression profile and track the entry of vectors in the cell. The *in vitro* assay was performed in HT-1080 using purified AAV2 viral vectors at a MOI of 1 × 10^4^ V.G./cell. The cells were analyzed by flow cytometry at eight different time points post-transduction to evaluate the percentage of GFP and mCherry positive cells (Figure 7; Supplementary Figure S2).

The results for GFP positive cells showed that all samples presented a similar transgene expression at 72 h post transduction. Despite different kinetic profiles, all vectors presented no GFP at 2 h post-transduction and its maximum expression (100% of positive cells) at 72 h post-transduction. VP1 C_587 was the insertion that presented the most delayed expression of the GFP transgene, compared to controls, even though it reached 95% at 72 h post transduction. In contrast VP2 E_138 presented the fastest GFP expression kinetics, similar to the respective control.

The activity of the mCherry present in the capsid of the AAV2 vectors was measured by the percentage of mCherry-positive cells. The results showed a more pronounced mCherry profile difference between conditions. More specifically, VP1 mCherry insertions presented almost half the mCherry signal than VP2 insertions at 48 h post-transduction. For VP2 D and E insertion sites, results showed positive cells (5%–10%) for mCherry as soon as 2 h post-transduction, while C_453 and C_587 only showed the same signal around 24 h. At 12 h post transduction D and E mutations already present 38% and 18%, respectively. This indicates a faster transduction profile that aligns with the earlier GFP transgene expression for the VP2 insertion strategies (D, E). For VP1 strategies (C), the mCherry peak was at 48 h, with almost 20% of mCherry-positive cells. This value was only achieved by insertion site 453. For VP2 strategies, D_138 presented the highest value at 24 h with around 75% of mCherry positive cells maintaining the signal at 48 h. Overall indicating up to 4-fold difference in mCherry signal between insertion sites C_453 and D_138. These results are also in accordance with the obtained in Figure 4B in the validation of the mCherry fusion constructs, where mCherry activity in residues 453 of VP1 (C) was shown to be up to 3-fold less than mCherry activity in residue D_138 and E_138. Overall, the strategy that presented the best mCherry signal and engineered particle transduction profile was strategy D followed by E, using residue 138 in VP2. The third best was C_453, and lastly, C_587 was the insertion that presented the lowest mCherry signal.

#### 3.2.5 AAV2/mCherry vectors biophysical characterization

**FIGURE 8:**
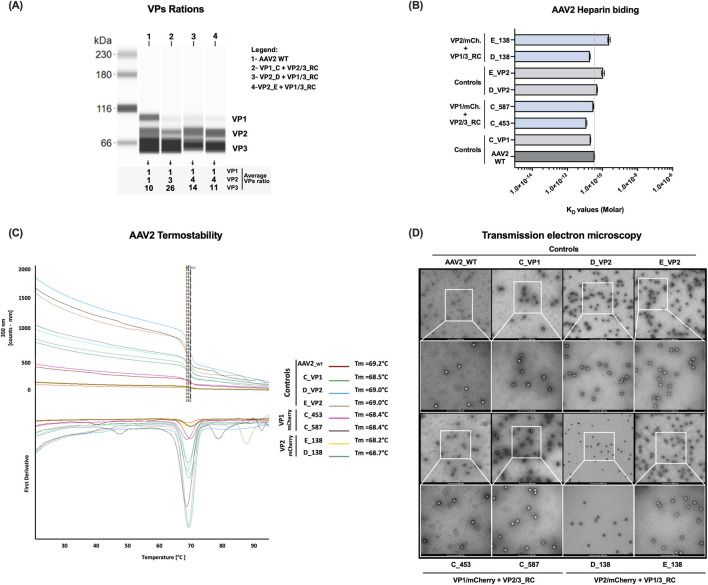
Evaluation of the biophysical properties of engineered AAV2/mCherry vectors. Two independent productions and purifications of the AAV2/mCherry engineered vectors were used to determine the biophysical properties. **(A)** Average VP1/2/3 ratios of mosaic control vectors by capillary western blot analysis by immunoblotting with anti-VP1/2/3 antibody VP1 (87 kDa), VP2 (72 kDa), VP3 (62 kDa) were detected by chemiluminescence channel. WB compass software 6.2.0 (ProteinSimple, CA, USA) was used for simulation of a standard polyacrylamide gel run. **(B)** Heparin binding by biolayer interferometry using Octet system, **(C)** Thermostability by nanoDSF using Prometheus system. **(D)** Capsid structural profile by transmission electron microscopy (Supplementary Figure S2) (scale bar 20 nm and 100 nm). C_VP1- control only expressing VP1; C_453 -Expresses VP1, with mCherry inserted into 453 site; C_587- Expresses VP1, with mCherry inserted into 587 site; D_138- Expresses VP2 (and VP3) with mCherry inserted into 138 site; E_138-Expresses VP2, with mCherry insertion on 138 site; VP2/3_RC- expresses VP2 and VP3 with *rep*; VP1/3_RC- expresses VP1 and VP3 with *rep*. All data represent the mean ± SD; *n* = 2.

To understand the behavior of mosaic and engineered AAV2/mCherry particles and the impact of different insertion sites, a comprehensive assessment of key biophysical properties is essential (Summerford and Samulski, 1998; Spink, 2008; Drouin et al., 2016). To achieve these, four distinct evaluations were conducted: viral vector protein stoichiometry was determined by capillary western blot analysis to quantify the amount of each VP present in the engineered vectors. AAV2 primary cell receptor [heparan sulfate proteoglycan (HSPG)] binding strength was assessed using the Octet system using heparin ligand. The thermostability profile of the engineered AAV2/mCherry particles was characterized using nano differential scanning fluorimetry. Transmission electron microscopy (TEM) images were analyzed to identify potential aggregates, impurities, and the structural integrity of the particles (Figure 8).

Capillary western blot Figure 8A results indicate that the VP1: VP2: VP3 ratios in the mosaic vectors deviated from those observed in the wild-type vector (1:1:10) (Schmit et al., 2019). The alteration in VP ratios correlated with the specific promoter used in the case of VP2 strategy, exhibiting a 4-fold increase in VP2. For the VP1 strategy, an increase in VP3 of 16-fold compared to the wild-type was observed. Receptor binding affinity assays revealed no differences between the engineered vectors and the wild-type control (Figure 8B). The observed equilibrium dissociation constant (K_D_) values, ranging from 1 × 10^11^ to 2.4 × 10^10^ (picomolar), suggest high-affinity interactions across all tested samples (Landry et al., 2012). While the VP2 strategy exhibited a marginally lower K_D_ value, this effect was attributed to the vector backbone rather than the mCherry insertion, as evidenced by similar behavior in the mosaic vector lacking the mCherry insertion. Figure 8C shows that the thermo-stability analysis reproduced the characteristic thermal profile of the AAV2 serotype and demonstrated that the engineered AAV2/mCherry particles closely resemble the wild-type control. nDSF data revealed that the melting temperature (Tm) values for all vectors ranged between 68.2 °C and 69.2 °C, suggesting that neither the mosaic capsid nor the protein insertion compromised the thermal stability of the particles. The TEM images indicated that the particles displayed uniformity in size and shape, with no aggregation or structural abnormalities detected (Figure 8D; Supplementary Figure S3). All vector preparations exhibited a homogenous distribution of particles, displaying an icosahedral form, and the particle size range for all samples was consistent with the expected dimensions for AAV particles. These comprehensive data demonstrate that while the development of the mosaic vectors altered the ratios of capsid proteins, it did not compromise vector’s structural integrity, thermal stability, or receptor binding affinity, even after the insertion of the mCherry protein.

## 4 Discussion

Despite being one of the vectors of choice for gene therapy, AAV vectors are still constantly being improved. Rational design strategies have been employed in capsid engineering to develop vectors with enhanced functionalities. One common method for engineering AAV capsids involves inserting peptides at specific locations. Although some large proteins have been introduced into the AAV capsid, this often leads to a significant reduction in both viral genome and transducing titers (Gigout et al., 2005). To address this issue, capsid mosaic approaches have been used to maintain the production of engineered AAV particles. While some studies suggest that the AAV2 capsid can accommodate proteins of various lengths, not all positions within the capsid are tolerant and flexible to large protein insertions (≥20 kDa) (Muralidhar et al., 1994). This work showed the versatility of different capsid insertion sites and the behavior of modified capsids upon insertion of a 28 kDa mCherry protein at residues 587, 453, or 138 (VP1 numbering), using a capsid mosaic approach. This ultimately provides a valuable benchmark for new AAV capsid designs that broaden the applications of these vectors to deliver large protein cargo.

The results presented in this work demonstrate that the AAV2 capsid can accommodate substantial mCherry insertions at the three tested sites without compromising its biophysical and biochemical integrity and particle functionality. As observed in Figures 2, 5, 8, alterations in particle titer or biophysical properties were associated with the development of mosaic vectors, rather than the insertion of the 28 kDa mCherry protein itself. This could be attributed to the inherited alterations in the cap expression cassette for the establishment of the mosaic vectors. To ensure robust expression of the isolated VPs, a more potent CMV promoter was employed in place of the native AAV p40 promoter (Fakultät and Teschner, 2020). The CMV promoter is widely recognised for its efficacy in enhancing recombinant gene expression in mammalian cells, resulting in a more pronounced expression of VP proteins compared to the WT (Moritz et al., 2015). This promoter substitution may account for the observed increase in the isolated VPS protein production (Figure 1C). Furthermore, the employment of a different promoter led to the disruption of the natural AAV2 stoichiometry of 1:1:10 in mosaic vectors (Figure 8A), with certain constructs exhibiting an increased presence of VP2 or VP3. Despite this disruption, no adverse effects on vector stability or assembly were noted, suggesting an inherent flexibility in the AAV2 capsid’s assembly requirements (Figures 8C,D). Nevertheless, these ratios are critical for the proper function of each viral protein in transduction. Analysis of the transducing units revealed an 8-fold reduction in titers for the mosaic vectors compared to AAV2 wild-type, potentially attributable to disruptions in the mosaic vector ratios. Despite the differences in the VP stoichiometry in the mosaic vectors, it did not compromise the overall quality of the developed mosaic vectors. Although large protein insertions into the AAV capsid could theoretically alter antibody accessibility in capsid-based ELISA assays, no evidence of impaired ELISA detection was observed in this study. Total particle titers remained comparable between the mosaic controls and the corresponding AAV2/mCherry engineered vectors, suggesting that mCherry insertion did not substantially interfere with AAV2 capsid recognition.

The integration of the mCherry protein into the viral proteins, irrespective of the insertion site, resulted in a decrease in the protein’s overall fluorescence. The insertion at site 587 in VP1 demonstrated the most significant effect on both mCherry activity and the functionality of the AAV2 vector. This observation suggests that the fusion of mCherry within this VP1 site may hinder its proper folding or lead to steric hindrance, thereby affecting its fluorescent properties and potentially compromising viral transduction. Modifications to the linkers adjacent to mCherry, or alterations of the amino acids to improve capsid compatibility and linker length (GGSG), could be considered for enhanced protein flexibility within the VP and assembled capsid. Conversely, insertions at sites 138 and 453, while still affecting mCherry fluorescence, preserved more of the protein’s activity, indicating these sites are more permissive for functional protein integration without severe structural or functional compromises.

Located on the exterior of the AAV capsid, the 453 insertion site in VP1 was found to be the second most flexible site for insertion of large cargo. This observation aligns with the findings of Boucas et al.^6^, who demonstrated that specific residue substitutions at position 453 conferred enhanced target receptor binding and cell transduction efficiency compared to those at position 587 (Boucas et al., 2009). Moreover, it is known that in AAV2 capsid proteins, a group of basic amino acids (arginines 585 and 588) contribute to heparin binding (Kern et al., 2003). Even though these insertion sites in VP1 were close to this region, no disruption in the heparin binding was observed (Figure 8B). However, when compared to the WT control, both VP1 insertion strategies exhibited delayed cellular entry, indicating an impact to some degree in particle transduction that varies with the insertion site. Although flow cytometry provided a sensitive and quantitative readout, future studies using optimized high-resolution microscopy approaches would be valuable to directly visualize engineered AAV vectors and further investigate intracellular particle trafficking.

Site 138 (both constructs D and E), while situated in a more internal region, demonstrated the highest flexibility and the least impact on mCherry activity and vector transduction among the three tested sites. The mCherry fusion at residue 138 (VP1 numbering) is situated in the N-terminal region of the VP2 protein, where the inherent flexibility of terminal regions likely accounts for the preserved structural integrity and functionality of mCherry (Bennett et al., 2023). Moreover, existing studies indicate that the VP2 capsid protein can accommodate large peptide insertions at the N-terminus, while other capsid sites exhibit lower tolerance (Warrington et al., 2004). Our results support these findings when comparing VP1 and VP2 insertion sites. While construct E_138 exclusively expresses VP2, D_138 only possesses a mutation at the VP1 start codon, leading to the expression of both VP2 and VP3. The VP3 protein is not only the most abundant in the AAV2 capsid but is also essential for particle assembly and capsid stability^13^. Therefore, the slight increase VP3 expression in mutation D_138 (Figure 8A) may contribute to the transduction profile observed with this strategy, thereby accounting for the difference in results between the two VP2 strategies (Figure 7B).

In summary, this study highlights the flexibility of AAV capsid insertion sites and the performance of modified capsids after incorporating large proteins. These findings suggest that although mosaic vectors can alter the AAV vector by modifying the VP configurations, subsequent engineering allows for the incorporation of large proteins without compromising their structural integrity or function. Residues 138 and 453 were identified as particularly permissive sites for such modifications. Overall, this work brings new insights for developing novel strategies and technologies in gene therapy, expanding the potential for AAV-based vectors to deliver larger protein payloads.

## Data Availability

The raw data supporting the conclusions of this article will be made available by the authors, without undue reservation.
