# Supplementary material for: AAV capsid sites breakdown: large protein insertions impact on vector dynamics
Source: Front Bioeng Biotechnol. 2026 Jul 3;14:1862682. doi: 10.3389/fbioe.2026.1862682 (PMC13375808; doi:10.3389/fbioe.2026.1862682)
Supplement: Supplementary file 1 [file Supplementaryfile1.docx]

Supplementary Material

# Supplementary Figures and Tables

#
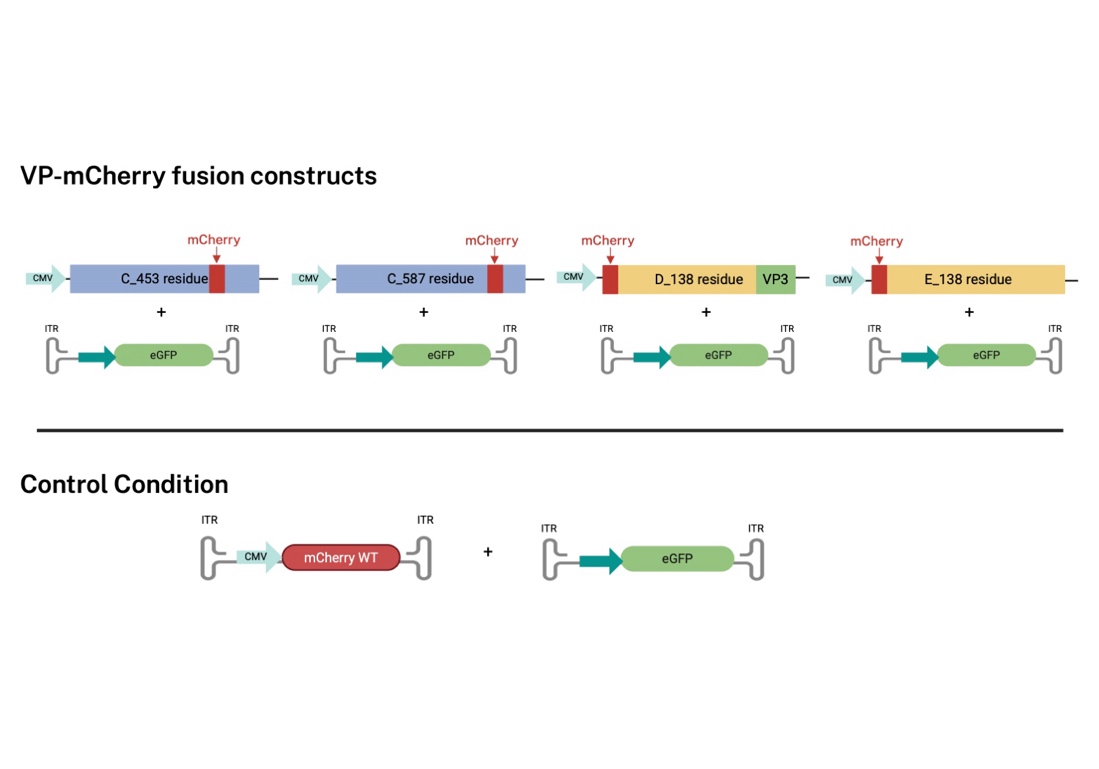


**Figure S1. Combination of plasmids for VP-mCherry fusion constructs validation**. A GFP-expressing plasmid was used as a transfection control. A mCherry-expressing plasmid was used for the WT mCherry control. The illustrations were created with BioRender.com accessed on the 15 May 2025.

**
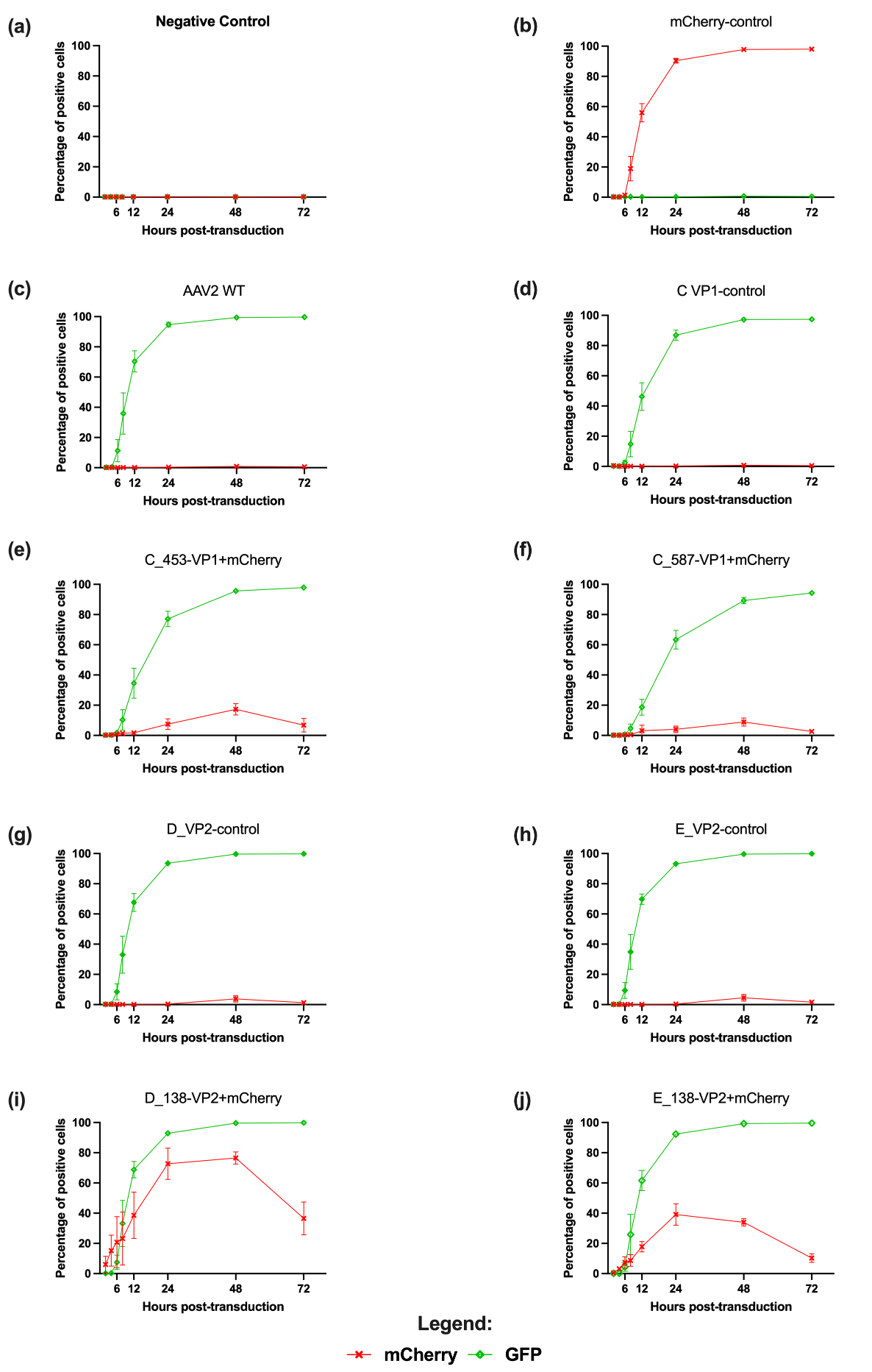
**

**Figure S2- Individual evaluation of the transduction profile of engineered AAV2/mCherry vectors. (A)** Negative control; (B) mcherry transgene used as control; (C) AAV2 RepCap wild type control; (D) VP1 C mutation control; (E) VP1, C mutation with mCherry inserted into 453 site; (F) VP1, C mutation with mCherry inserted into 587 site; (G) VP2_D_mutation control; (H) VP2_E_mutation control; (I) VP2_D_mutation on 138 site; (J) VP2_E_mutation on 138 site. Purified AAV2 vectors were used to transduce HT1080 cells at a vector dose of 1x10^4^ V.G./cell. The results were evaluated by flow cytometry at various time points: 2, 4, 6, 8, 12, 24, 48 and 72hours post-transduction. Green bars correspond to expression of eGFP and red bars correspond to expression of mCherry protein, fused to the AAV2 capsid.


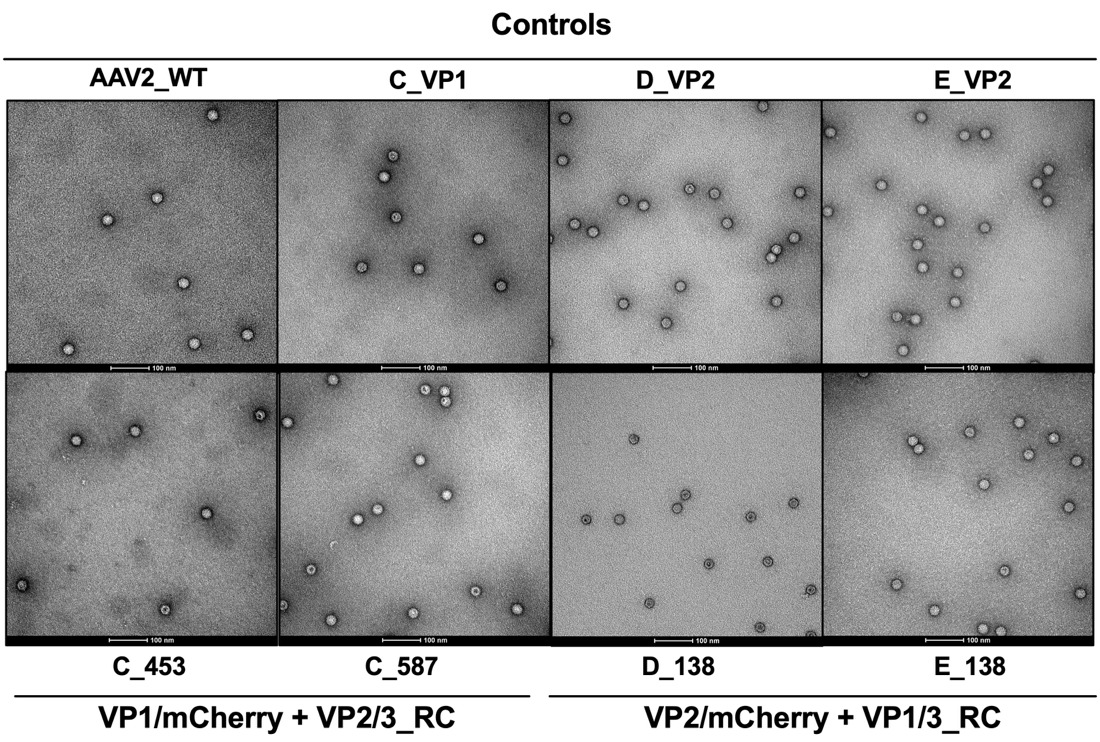


**Figure S3. Biophysical properties of engineered AAV2/mCherry vectors:** **capsid structural profile by TEM.** Capsid structural profile by transmission electron microscopy (scale bar 100 nm). C_VP1- control only expressing VP1; C_453 -Expresses VP1, with mCherry inserted into 453 site; C_587- Expresses VP1, with mCherry inserted into 587 site; D_138- Expresses VP2 (and VP3) with mCherry inserted into 138 site; E_138-Expresses VP2, with mCherry insertion on 138 site; VP2/3_RC- expresses VP2 and VP3 with rep/cap; VP1/3_RC- expresses VP1 and VP3 with rep/cap.

| **Strategy** | | **Missense Mutation** | **Reference** |
| --- | --- | --- | --- |
| **VP1 isolation strategy** | **A** | Major splicing acceptor (nt 2228)- (Asp to Tyr)- This This abolishes the expression VP2 and VP3. | 1,2 |
|  | **B** | Start Condon of VP2 (T138A) and VP3 (M203L + M211L + M235L)- This abolishes the expression of VP2 and VP3. |  |
|  | **C** | Major splicing acceptor (nt 2228- (Asp to Tyr) and start Condon of VP2 (T138A) and VP3 (M203L + M211L + M235L). |  |
| **VP2 isolation strategy** | **D** | Start Condon of VP1 (M1L) - This abolishes the expression of VP1. |  |
|  | **E** | Start Condon of VP1 (M1L) and VP3 (M203L + M211L + M235L)- This abolishes the expression of VP1 and VP3. |  |
| **Wild-type rep/cap complementation cassettes** | **VP2/3_RC** | Start Condon of VP1 (M1L)- This abolishes the expression of VP1. |  |
|  | **VP1/3_RC** | Start Condon of VP2 (T138A)- This abolishes the expression of VP2. |  |

**Table S1. Mosaic vector development.** Missense mutations and developed constructs.

1- Grosse, S., Penaud-Budloo, M., Herrmann, A., Börner, K., Fakhiri, J., Laketa, V., Krämer, C., Wiedtke, E., Gunkel, M., Ménard, L., Ayuso, E., & Grimm, D. (2017). Relevance of assembly-activating protein for adeno-associated virus vector production and capsid protein stability in mammalian and insect cells. Journal of Virology, 91, e01198-17. https://doi.org/10.1128/jvi.01198-17

2- Guo, P., El-Gohary, Y., Prasadan, K., *et al.* (2012). Rapid and simplified purification of recombinant adeno-associated virus. Journal of Virological Methods, 183(2), 139–146. https://doi.org/10.1016/j.jviromet.2012.04.004
